# Analysis of the Transcriptome Response to Low Nitrogen in *Populus ussuriensis*

**DOI:** 10.3390/biology14101448

**Published:** 2025-10-20

**Authors:** He Feng, Yue Chang, Runze Liu, Wenlong Li, Zhiwei Liu, Ming Wei, Zhibin Luo, Chenghao Li

**Affiliations:** 1 State Key Laboratory of Tree Genetics and Breeding, Northeast Forestry University, Harbin 150040, China; 2020023050@nefu.edu.cn (H.F.); 15131534900@163.com (Y.C.); liurunze@nefu.edu.cn (R.L.); 2021111160@nefu.edu.cn (Z.L.); weiming0312@nefu.edu.cn (M.W.); 2 School of Forestry, Northeast Forestry University, Harbin 150040, China; 3 College of Environmental and Resource Sciences, Dalian Minzu University, Dalian 116600, China; wenlongli@dlnu.edu.cn; 4 State Key Laboratory of Tree Genetics and Breeding, Research Institute of Forestry, Chinese Academy of Forestry, Beijing 100091, China; luozbbill@163.com

**Keywords:** low nitrogen stress, *Populus ussuriensis*, root, real-time sequencing, regulatory network

## Abstract

**Simple Summary:**

Nitrogen is a vital nutrient that forest trees absorb from the soil through their roots. A lack of nitrogen greatly reduces the growth of forest trees. In this study, we aim to understand how wild-type *Populus ussuriensis* plantlets grown in vitro respond to low-nitrogen conditions at the molecular level. We grew *Populus ussuriensis* under both normal and low-nitrogen conditions and observed how they developed over time. Our results showed that when nitrogen was limited, *Populus ussuriensis* grew more roots but produced less shoot tissue. Bioinformatics analyses of root gene activity revealed over 8000 genes that exhibited altered expression patterns under nitrogen stress. These genes primarily regulate nitrogen uptake and utilization, antioxidant defense, root development, and internal hormone regulation. Among them, 443 transcription factors (mainly of the MYB and AP2/ERF types) were identified among all differentially expressed genes. Additionally, we constructed a regulatory network comprising 60 nitrogen-metabolism-related genes that collectively coordinate the nitrogen stress response in *Populus ussuriensis*. This study establishes a systematic foundation for investigating molecular adaptation mechanisms in *Populus ussuriensis* roots under nitrogen stress.

**Abstract:**

(1) Background: Nitrogen is a key element that is essential for plant growth, and it is absorbed by roots from the soil. Nitrogen stress severely limits forest tree productivity; therefore, elucidating the molecular mechanisms underlying nitrogen stress tolerance in forest trees is critical for sustainable forestry. (2) Methods: Phenotypic analyses of wild-type (WT) *Populus ussuriensis* (*P. ussuriensis*) plantlets grown in vitro were carried out at different time points under both normal and low-nitrogen conditions. Transcriptome analyses of roots were performed at 0, 12, 24, 48, 96, and 336 h under low-nitrogen stress via RNA-seq. A gene regulatory network (GRN) for nitrogen-metabolism-associated DEGs was constructed using a three-gene module framework and a bottom-up Gaussian Graphical Model algorithm. (3) Results: WT *P. ussuriensis* plantlets grown in vitro exhibited a synergistic response characterized by increased root biomass and suppressed shoot growth. Transcriptome analyses identified 8289 DEGs enriched in nitrogen metabolism, ROS scavenging, root development, and phytohormone signaling. A total of 443 differentially expressed transcription factors (TFs) (mainly MYB, AP2/ERF, and bHLH) were detected. A nitrogen-metabolism-associated GRN comprising 60 nodes was established. (4) Conclusions: Transcriptomic data and nitrogen metabolism pathway predictions from this study establish a systematic foundation for investigating molecular adaptation mechanisms in *P. ussuriensis* roots under nitrogen stress.

## 1. Introduction

Nitrogen (N) is an essential macronutrient for plant growth and development, accounting for approximately 1.5–2% of plant dry weight [1]. It is a fundamental constituent of structural and functional macromolecules, including proteins, nucleic acids, and enzymes [2], and plays a crucial role in maintaining normal metabolic processes related to energy and matter in plants. Plants primarily absorb and utilize inorganic N [3], with ammonium (NH_4_^+^) and nitrate (NO_3_^−^) as the main forms [4]. The ratio between NH_4_^+^ and NO_3_^−^ concentrations significantly influences plant growth and development, in addition to the uptake of other nutrients [5]. Notably, NO_3_^−^ acts as a signaling molecule, regulating the expression of nitrate transporter (NRT) genes through feedback mechanisms [6,7]. Insufficient N availability impairs N absorption and metabolism, hindering the synthesis of N-containing compounds such as proteins, amino acids, and nucleic acids [8] and ultimately affecting plant morphology, physiology, and yield.

Soil N levels are generally low due to natural and anthropogenic factors, including vegetation, climate, topography, soil texture, and microbial activity [9]. To cope with N limitation, plants enhance both internal nitrogen use efficiency (NUE) and external N acquisition capacity by remodeling the root system’s architecture [10]. Common morphological adaptations include primary root elongation, increased lateral root branching, and enhanced root hair development, collectively increasing the root’s absorptive surface area [11]. The MADS-box transcription factor *ANR1* promotes lateral root elongation and serves as a key regulator of local nitrate sensing [12]. These changes exhibit differential responses to N availability: Mild deficiency promotes root elongation, while severe deficiency inhibits primary root growth and suppresses lateral root formation [13,14]. In addition, *OsNAR2.1* mediates the nitrate signaling pathway, thereby regulating lateral root development [15]. Similarly, under N stress, the overexpression of *GmGLP20.4* enhances soybean root biomass by improving root architecture; transgenic tobacco studies further confirm that overexpression promotes plant growth and optimizes root architecture [16]. Root plasticity is crucial for plant nutrient uptake and adaptation to changing environmental conditions [17].

NO_3_^−^, the dominant form of N available to plants, is taken up via NO_3_^−^ transporters, primarily those from the *NRT1/NPF* and *NRT2* families. The overexpression of phytoglobin in *Oryza sativa* (*O. sativa*) enhances the expression of *OsNRT2.1*, *OsNRT2.3*, and *OsNRT2.4*, which promotes growth and nitrate uptake under N-deficient conditions [18]. Likewise, transgenic maize lines overexpressing *ZmNRT2.1* or *ZmNRT2.5* exhibit enhanced root nitrate uptake and growth under LN conditions [19]. In addition to NO_3_^−^, NH_4_^+^ constitutes another essential inorganic N source that is directly absorbed by plants. The overexpression of ammonium transporter *AMT1;1* in *Arabidopsis thaliana* (*A. thaliana*) and *O*. *sativa* increases ammonium uptake and improves NUE under limited N supply [20,21]. Following uptake, N is rapidly assimilated into organic compounds through a core metabolic network involving nitrate reductase (NR), nitrite reductase (NiR), glutamine synthetase (GS), and glutamate synthase (GOGAT), which convert inorganic N into amino acids. The overexpression of genes encoding these enzymes, such as *GS1;1* or *NADH-GOGAT*, enhances N assimilation and growth in multiple species, including *O. sativa* and *Panax notoginseng* [22,23]. These results indicate that plants may enhance the activities of key enzymes involved in N uptake and assimilation, thereby optimizing N source acquisition and its utilization efficiency and ultimately improving plant adaptability to N-limited environments.

Nitrogen limitation also induces oxidative stress, resulting in the accumulation of reactive oxygen species (ROS) such as hydrogen peroxide (H_2_O_2_) and superoxide anions (O_2_^−^), which impair plant growth and may lead to death [24]. Under LN conditions, wheat activates its antioxidant defense system and enhances ROS scavenging capacities by significantly upregulating peroxidase (POD) gene expression in roots [25]. Although these adaptive responses are well characterized in model herbaceous species, the underlying regulatory mechanisms in woody perennials, such as *Populus ussuriensis* (*P. ussuriensis*), remain largely unknown.

Phytohormone signaling pathways mediate the coordination of root development and NUE in plants under LN conditions, thereby enhancing their adaptability to N-deficient conditions [26]. In *A. thaliana*, LN stress upregulates the expression of the auxin biosynthetic gene *TAR2*, promoting auxin accumulation and stimulating lateral root formation. This process remodels the root architecture to improve N acquisition efficiency [27]. Additionally, LN conditions activate abscisic acid signaling mediated by the LATD/NRT1 transporter, suppressing lateral root initiation while promoting primary root elongation. This dual response enables the root system to effectively adapt to the spatially heterogeneous N availability in the soil [28]. Furthermore, LN stress triggers the ethylene signaling pathway, which negatively regulates nitrate reductase NR activity through the CTR1-EIN2 cascade. This inhibition of nitrate assimilation fine-tunes the plant’s adaptive response to N limitation [29]. Collectively, these findings demonstrate that phytohormone-mediated root plasticity is crucial for efficient N acquisition and environmental adaptation.

Transcription factors (TFs) play critical regulatory roles in plant responses to N deficiency, particularly during N availability adaptation. Distinct classes of TFs perform specialized functions, collectively forming a sophisticated transcriptional regulatory architecture. During N acquisition, *AtTGA1* and *AtTGA4* enhance uptake efficiency by promoting lateral root development in *A. thaliana*. While the roles of *AtTGA1* and *AtTGA4* in enhancing nitrogen uptake are well-established in *A. thaliana*, it remains unclear whether their functional homologs in woody perennial species such as *P. ussuriensis* employ a conserved or divergent regulatory strategy under nitrogen limitations. In contrast, *MdbHLH130* achieves this process in apple rootstocks by modulating relevant metabolic fluxes [30,31]. Following absorption, *MdDREB2A* facilitates N assimilation through the activation of key genes, establishing the material basis for plant growth [32]. Beyond assimilation, *AtWRKY1* integrates light signaling and N signaling pathways to optimize carbon–nitrogen resource allocation [33]. Furthermore, under LN conditions, the LBD TFs in *A. thaliana* enhance plant adaptability by regulating nitrate signaling transduction and stress responses [34]. These TF families function synergistically, collectively ensuring plant efficiency in N utilization and environmental adaptation.

In this study, we integrated transcriptomic data with a focus on N metabolism pathway genes and constructed a gene regulatory network (GRN) based on a tri-gene module framework. This GRN also includes the members of the aforementioned bZIP, AP2/ERF, and WRKY TF families. These genes all play a role in the N metabolism pathway by regulating the functional genes related to N absorption, transport, and assimilation, thereby revealing the spatiotemporal regulatory mechanisms underlying plant adaptation to LN conditions.

## 2. Materials and Methods

### 2.1. Plant Material and Treatments

Clonally propagated *P. ussuriensis* plantlets were maintained in vitro on Woody Plant Medium (WPM) [35] supplemented with 25 g/L sucrose and 6 g/L agar. The pH was 5.8–6.0, and the medium was autoclaved at 121 °C for 15 min. Cultures were incubated under a 16 h light/8 h dark photoperiod at 23 °C, with a light intensity of 46 μmol photons m^−2^ s^−1^ provided by cool white fluorescent lights. The upper three internodes were excised from 4-week-old plantlets and transferred via micro-cutting to normal nitrogen (NN) WPM, which contained 10 mM NO_3_^−^ and 5 mM NH_4_^+^. After three weeks of rooting, the rooted plantlets were subjected to LN conditions by transferring them to modified WPM containing 0.05 mM NO_3_^−^ and 0.025 mM NH_4_^+^ [36]. Root samples were collected at 0, 12, 24, 48, 96, and 336 h after LN. All samples were rapidly frozen in liquid N and subsequently stored at −80 °C for RNA-seq.

### 2.2. Morphology and Growth Trait Measurement of Wild-Type P. ussuriensis Under Normal Nitrogen and Low-Nitrogen Stress

The WT *P. ussuriensis* in vitro plantlets treated under NN and LN conditions were sampled at 0, 12, 24, 48, 96, and 336 h. Each treatment included 15 biologically independent replicates. At each time point, representative photographs were taken, and morphological traits were documented, including shoot fresh weight, root fresh weight, and plant height. The quantitative data derived from these measurements were used to generate column graphs, which were plotted using GraphPad Prism 8.0 (GraphPad Software, San Diego, CA, USA). To statistically evaluate the morphological effects of LN stress on plant development over time, the data obtained at each time point were subjected to Student’s *t*-test using SPSS for Windows, Version 16.0 (SPSS Inc., Released 2007. Chicago, IL, USA). This statistical method was selected because it is ideally suited for comparing the means between two independent groups (NN vs. LN at each respective time point), allowing for a direct assessment of the treatment effect under the assumption that the data meet the test’s prerequisites. These measurements were used to assess the morphological effects of LN on plant development over time.

### 2.3. RNA-Seq Experiment, Library Construction, and Sequencing

The CTAB (cetyltrimethyl ammonium bromide) method was used to extract total RNA from samples. This protocol, based on a previously described CTAB-based approach for plant total RNA isolation, enables highly efficient extraction of high-quality RNA from tissues rich in polysaccharides and polyphenols of grapevine and various other woody plant species [37]. Library construction and sequencing were performed by Annoroad Gene Technology Corp (Beijing, China). RNA concentration and purity were measured using a Nano Photometer^®^ spectrophotometer (IMPLEN, Westlake Village, CA, USA). RNA integrity was assessed using RNA Nano 6000 Assay Kit (Agilent Technologies, Santa Clara, CA, USA) with the Agilent Bioanalyzer 2100 system (Agilent Technologies, Palo Alto, CA, USA). A total amount of 1 µg total RNA per sample was used as input material for RNA library preparations.

Sequencing libraries were generated using the NEB Next^®^Ultra™ RNA Library Prep Kit for Illumina^®^ (NEB, Ipswich, MA, USA) following the manufacturer’s recommendations. Index codes were added to attribute sequences for each sample. The libraries were sequenced on an Illumina platform (Illumina HiSeq 2500) (Illumina, Inc., San Diego, CA, USA), generating paired-end reads. Raw reads were processed by removing reads containing adapters, reads containing poly-N, and low-quality reads to obtain clean reads. Quality metrics, including Q20, Q30, GC content, and sequence duplication levels, were assessed for the clean data. All downstream analyses were based on high-quality clean data. The clean reads were mapped to the genome of the *P. trichocarpa* reference genome for subsequent analyses.

### 2.4. Sequence Alignment to the Reference Genome and Expression Estimation

Since both *P. ussuriensis* and *P. trichocarpa* belong to the section *Tacamahaca*, their sequence similarity is high. Tacamahaca, a section of the genus Populus that includes species such as *P. trichocarpa* and *P. ussuriensis*, shares a recent “salicoid duplication” event with willows (*Salix*), which underlies their high degree of genetic and evolutionary similarity and establishes them as ideal models for studying genome evolution in woody plants [38]. Notably, the genome of *P. trichocarpa* exhibits extremely high allele consistency (99.74%), as reported in the same study. Based on this finding, we infer that the genomic similarity between the closely related species *P. ussuriensis* and *P. trichocarpais* is likely to approach or even exceed this value, further supporting their use in comparative genomic analyses within this evolutionarily significant group. The reference genome and annotation files were downloaded from NCBI (https://www.ncbi.nlm.nih.gov/datasets/genome/, accessed on 16 March 2025). Bowtie2 and TopHat2 were used to align the sequenced clean data to the reference genome. FPKM (fragments per kilobase of exon model per million mapped fragments) is a measure of gene expression, representing the number of fragments per kilobase of gene length per million mapped fragments. This method eliminates the influence of gene length and sequencing depth on gene expression quantification, enabling direct comparisons of gene expression differences between samples. The method’s formula is as follows: FPKM = total exon Fragments/(mapped reads (Millions) × exon length (KB)).

### 2.5. Differential Expression Analysis and Functional Annotation

Differentially expressed genes (DEGs) between each time point under LN conditions and the 0 h control were identified using the DESeq2 R package 1.48.1 [39]. Genes with |log_2_(fold change)| ≥ 1 and *q* value < 0.05 were classified as DEGs. The DEGs were functionally annotated using NCBI, Uniprot, and the GO and KEGG databases to obtain detailed functional descriptions.

Based on the upregulated and downregulated DEGs identified in different treatments, GO analysis was performed across three categories, including MF, CC, and BP. The GO annotations for genes or proteins can be assigned via ID or sequence annotation. These annotations link GO numbers to their corresponding terms. These terms correspond to functional categories or cell localizations, enabling the identification of significantly overrepresented biological functions.

### 2.6. Quantitative Real-Time Reverse Transcription-PCR

Total RNA was isolated using the CTAB method, and cDNA was synthesized using the cDNA Synthesis SuperMix Kit (TransGen Biotech, Beijing, China). Gene-specific primers for 12 randomly selected genes from the transcriptome sequencing data were designed using PRIMER PREMIER 5.0. The *Actin* gene, encoding a 42 kDa cytoskeletal protein, was utilized as an internal control for normalization. The 12 genes were *CYP81D8* (Potri.005G143800), *MYB111* (Potri.014G122700), *MLP423* (Potri.011G026100), *BGLU17* (Potri.001G222800), *CYP87A2* (Potri.009G064900), *ALD1* (Potri.002G091500), *CEL2* (Potri.001G083200), *WBC11* (Potri.001G311300), *RLK* (Potri.009G035400), *SCL21* (Potri.005G143900), *TPS-CIN* (Potri.019G023000), and *RNS1* (Potri.008G086700). RT-qPCR was performed using TransStart Top Green qPCR SuperMix (TransGen Biotech, Beijing, China) with a qTOWER 3G Cycler and qPCR software 4.1 (Analytik Jena, Analytik Jena, TransGen Biotech, Beijing, China); three biological replicates were used per gene. Quantitative data were analyzed using the 2^−△△Ct^ method [40]. All primers are listed in Table S8.

### 2.7. Regulatory Network of Transcription Factors in P. ussuriensis Under Low-Nitrogen Conditions

This study applied a three-gene model and a bottom-up GGM algorithm to construct the GRN for DEGs [41]. The gene regulatory network was pruned using TF-target binding data from PlantTFDB 5.0 (https://planttfdb.gao-lab.org/, accessed on 28 May 2025) to construct a four-layer GRN. The network visualization was performed using Cytoscape v3.10.3.

## 3. Results

### 3.1. Analysis of Growth Morphology Under Normal Nitrogen and Low-Nitrogen Conditions

**Figure 1:**
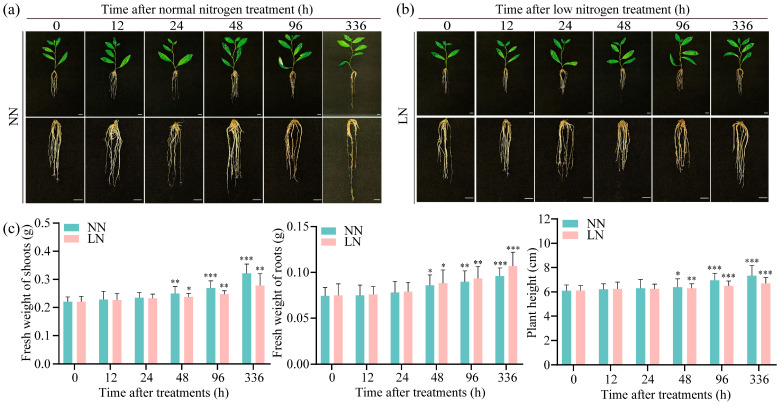
Analysis of growth morphology under normal nitrogen (NN) and low-nitrogen (LN) conditions. (**a**) The wild-type (WT) in vitro plantlets of *Populus ussuriensis* (*P. ussuriensis*) under NN conditions at 0, 12, 24, 48, 96 and 336 h, respectively, the scale bar is 1 cm; (**b**) The WT in vitro plantlets of *P. ussuriensis* under LN conditions for 0, 12, 24, 48, 96 and 336 h, respectively, the scale bar is 1 cm; (**c**) The bar charts comparing fresh weight of shoots, fresh weight of roots and plant height of the WT in vitro plantlets under NN and LN conditions at 0, 12, 24, 48, 96 and 336 h. Green bars represent NN, and pink columns represent LN conditions. Each treatment used 15 plants. *, ** and *** represent significant differences between LN and the NN conditions determined by Student’s *t*-test at *p* < 0.05, *p* < 0.01 and *p* < 0.001, respectively.

To investigate morphological changes in *P. ussuriensis* under LN conditions, 3-week-old in vitro plantlets were transferred to either normal or LN liquid media. After this step, the plants were transferred to normal and LN liquid culture media and then cultured for 0 h, 12 h, 24 h, 48 h, 96 h, and 336 h while monitoring growth status (Figure 1a,b). The results indicated no significant differences in shoot fresh weight, root fresh weight, or plant height between treatments during the first 24 h. After 48 h of LN conditions, root growth exceeded the results occurring under normal conditions with a 3% increase in biomass, while aboveground growth was suppressed, as shown by a 5% decrease in shoot fresh weight and a 1% reduction in plant height. After 96 h of LN conditions, root biomass increased by 4% compared with normal conditions, whereas above-ground growth further declined with an 8% decrease in shoot fresh weight and 7% reduction in plant height. After 336 h of LN conditions, root growth significantly improved with a 12% biomass increase over controls, while above-ground parts exhibited a 14% decrease in shoot fresh weight and an 8% reduction in plant height (Figure 1c).

### 3.2. Transcriptome Sequencing and Annotation

Root samples from wild-type (WT) *P. ussuriensis* in vitro plantlets under LN conditions were collected at 0, 12, 24, 48, 96, and 336 h, with 3 biological replicates per time point (18 total samples). Transcriptome sequencing was performed using the Illumina platform (Zhejiang Annord Biotechnology Co., Ltd, Jinhua, China.). Raw reads were filtered to generate high-quality clean reads, with >93% achieving Q30 scores. Approximately 90% of reads were aligned to the reference genome (*P. ussuriensis*; Tables S1 and S2).

**Figure 2:**
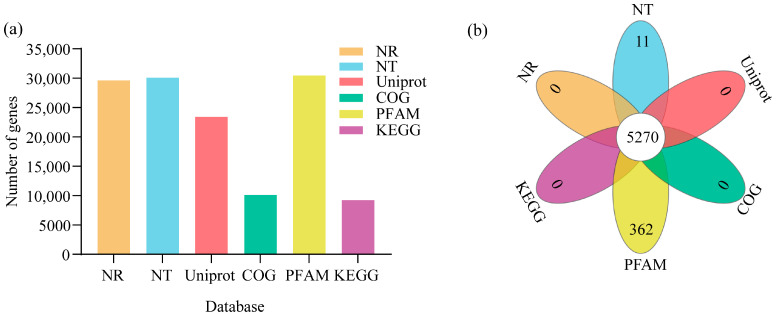
Transcriptome sequencing and annotation. (**a**) The number of gene annotations in different databases. The bar chart illustrates the number of genes annotated in the six databases, Sequence Database (Nr), Nucleotide Sequence Database (Nt), Universal Protein Database (Uniprot), Cluster of Orthologous Groups of proteins (COG), Protein family and domain classification database (Pfam), and Kyoto Encyclopedia of Genes and Genomes (KEGG). The x-axis labels the different databases, and the y-axis shows the number of genes annotated. (**b**) The Venn illustrates overlapping gene annotations across the six databases, NR, NT, UniProt, COG, PFAM, and KEGG.

Non-redundant genes were annotated against the NCBI Non-Redundant Protein Sequence Database (Nr); Nucleotide Sequence Database (Nt); Universal Protein Database (Uniprot); Cluster of Orthologous Groups (COG); Protein Family and Domain Classification Database (Pfam); and Gene Ontology (GO) and Kyoto Encyclopedia of Genes and Genomes (KEGG) databases (E-value < 1× 10^−5^). Among 30,485 unigenes, 29,607, 30,092, 23,403, 10,101, 30,429, 22,512, and 9221 were annotated in NR, NT, UniProt, COG, Pfam, GO, and KEGG, respectively. A total of 5270 genes were annotated in all six of these databases (Figure 2a,b).

### 3.3. Identification and Analysis of DEGs

**Figure 3:**
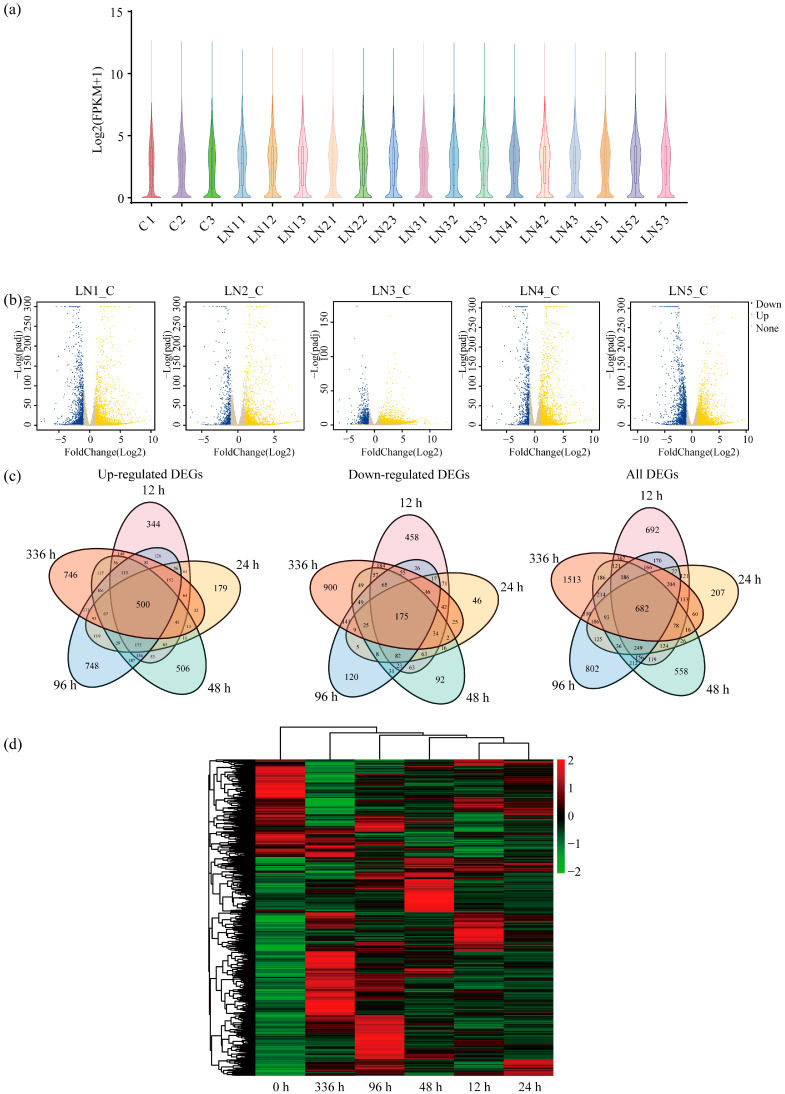
Identification and analysis of differentially expressed genes (DEGs) under normal nitrogen (NN) and low-nitrogen (LN) conditions. (**a**) The violin plot shows the distribution of gene expression. The x-axis indicates sample names, including three biological replicates for the control (C1-C3) and LN conditions at different time points (LN11-LN13 for 12 h, LN21-LN23 for 24 h, LN31-LN33 for 48 h, LN41-LN43 for 96 h, LN51-LN53 for 336 h), and the y-axis indicates the log_2_(FPKM + 1) values. The width of the violin represents gene density. (**b**) Volcano plots displaying DEGs for the five biological replicates (LN1_C to LN5_C, representing LN condition for 12, 24, 48, 96 and 336 h versus the control). The x-axis represents the log_2_(fold change) in gene expression, and the y-axis represents the −log_10_(*p*-value). Yellow dots denote upregulated genes, blue dots denote downregulated genes, and grey dots denote non-significant genes. (**c**) The Venn diagram analysis of upregulation, downregulation, and all DEGs at different time points (12, 24, 48, 96, and 336 h) under LN conditions versus control (0 h). (**d**) The heatmap of hierarchical clustering analysis of DEGs under LN conditions at different time points (12, 24, 48, 96, and 336 h). The x-axis indicates time points, and the y-axis represents genes. Red indicates upregulated expression and green indicates downregulated expression.

To visualize gene expression distributions across treatment groups, violin plots of the three control group samples revealed consistent expression patterns within this group. At the early LN time points of 12 h and 24 h, the suppression of some gene expression began. As stress duration increased, the 48 h and 96 h groups exhibited a further decrease in overall expression levels with an increase in dispersion. By the 336 h time point, gene expression stabilized into a relatively consistent pattern; although overall levels remained lower than the control group, the distribution became more regular and concentrated (Figure 3a).

Gene expression displays tissue and spatiotemporal specificity. DESeq2 identified differentially expressed genes (DEGs) (|log_2_FC| ≥ 1, q-value < 0.05). Volcano plots depicted these differential expressions (Figure 3b). Analyses identified 8289 DEGs between LN conditions and the control groups, comprising 5544 upregulated and 2979 downregulated genes (Figure 3c). Specifically, 3111 genes were upregulated at 96 h, and 2757 genes were upregulated at 336 h, with 500 genes upregulated across all time points. Downregulated genes numbered 1858 at 336 h and 1461 at 12 h; only 175 genes were downregulated throughout all time points. A heatmap visualized dynamic expression patterns across different LN conditions durations (Figure 3d).

**Figure 4:**
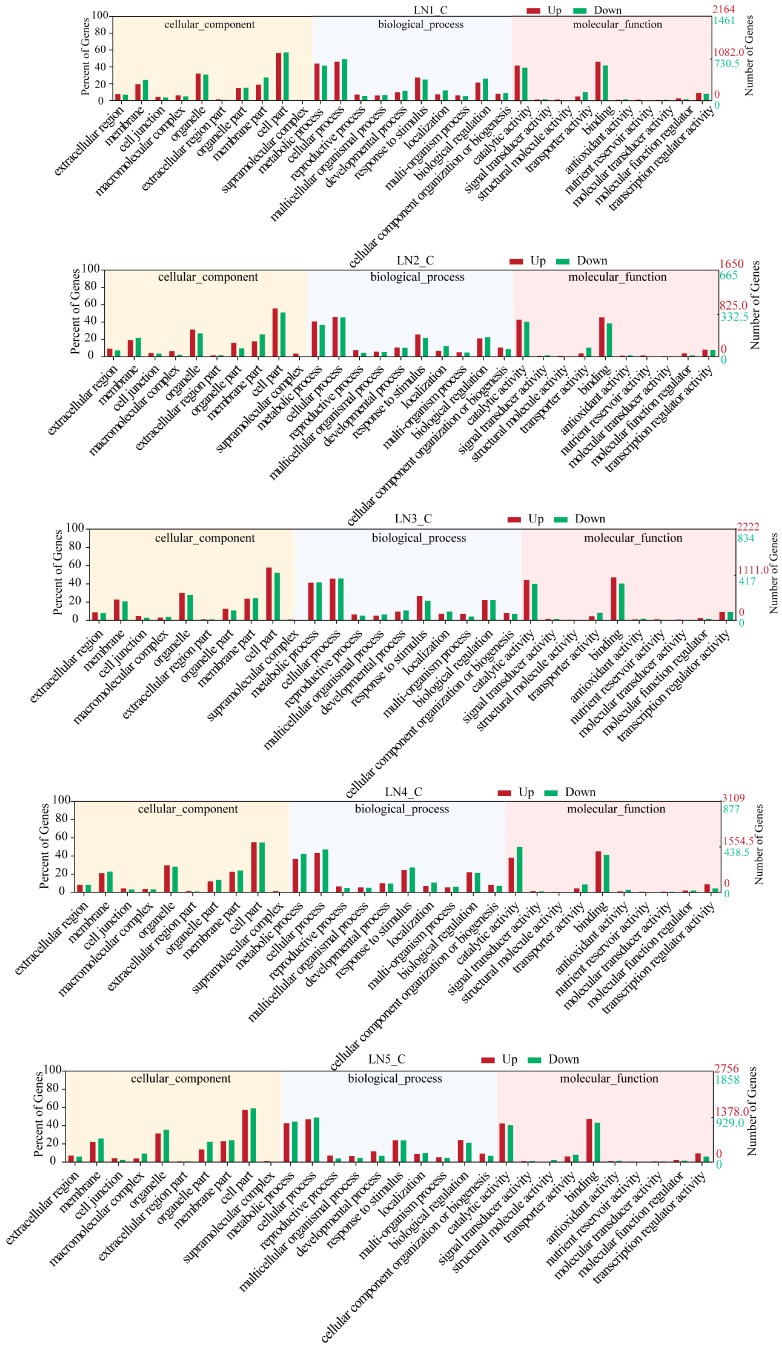
Gene Ontology (GO) enrichment analysis of wild-type (WT) plants under low-nitrogen (LN) conditions at different time points. The figure shows the distribution of differentially expressed genes (DEGs) at five different stress time points (LN1_C to LN5_C, representing LN condition for 12, 24, 48, 96 and 336 h versus the control) in three GO categories: Cellular Component, Biological Process, and Molecular Function. The left y-axis shows the percentage of DEGs relative to the total DEGs count. The right y-axis shows the number of DEGs. Red bars represent the percentage (left y-axis) and quantity (right y-axis) of the upregulated DEGs. Green bars represent the percentage (left y-axis) and quantity (right y-axis) of downregulated DEGs. Background colors distinguish the three GO categories.

**Figure 5:**
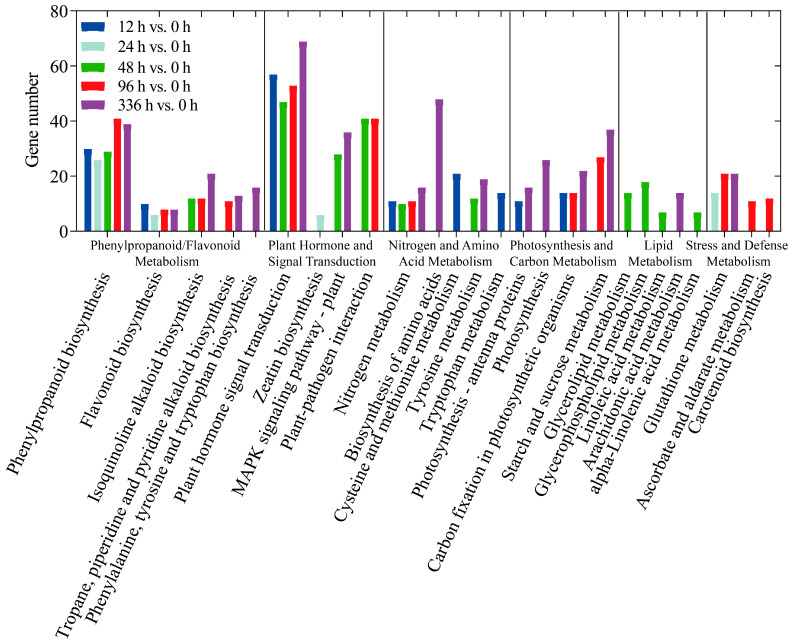
The classification of differentially expressed genes (DEGs) based on Kyoto Encyclopedia of Genes and Genomes (KEGG) pathways in transcriptome analysis. The x-axis represents KEGG metabolic pathways, which are grouped by their biological functions for clearer interpretation, such as Phenylpropanoid/Flavonoid Metabolism and Plant Hormone and Signal Transduction, and the y-axis represents the number of DEGs. The bars represent the distribution of DEGs under low-nitrogen conditions at different time points (12, 24, 48, 96 and 336 h) compared to the control group (0 h), with the following color code: dark blue for 12 h versus 0 h, light green for 24 h versus 0 h, green for 48 h versus 0 h, red for 96 h versus 0 h, and purple for 336 h versus 0 h.

Gene Ontology (GO) categorizes gene functions into three classes: Biological Process (BP), Cellular Component (CC), and Molecular Function (MF). Enrichment analysis revealed the primary biological functions of DEGs. The top three GO terms within each domain were as follows: CC, including cell part, organelle, and membrane part; BP, including cellular process, metabolic process, and response to stimulus; MF, including binding, catalytic activity, and transcription regulator activity (Figure 4). A total of 1213 DEGs were mapped to 125 Kyoto Encyclopedia of Genes and Genomes (KEGG) pathways. When these pathways were grouped by their biological functions for clearer interpretation (Figure 5), distinct temporal enrichment patterns emerged under LN conditions (*p* < 0.05). The enrichment was predominantly observed during the later stages (48 h, 96 h, and 336 h), with some pathways also being enriched at 12 h. Notably, pathways within the phenylpropanoid/flavonoid biosynthesis cluster (including phenylpropanoid biosynthesis and flavonoid biosynthesis) were significantly enriched across all or most time points. Similarly, the functional clusters of plant hormone signal transduction and nitrogen metabolism were significantly enriched at all time points except 24 h.

### 3.4. Nitrogen-Metabolism-Related DEGs

**Figure 6:**
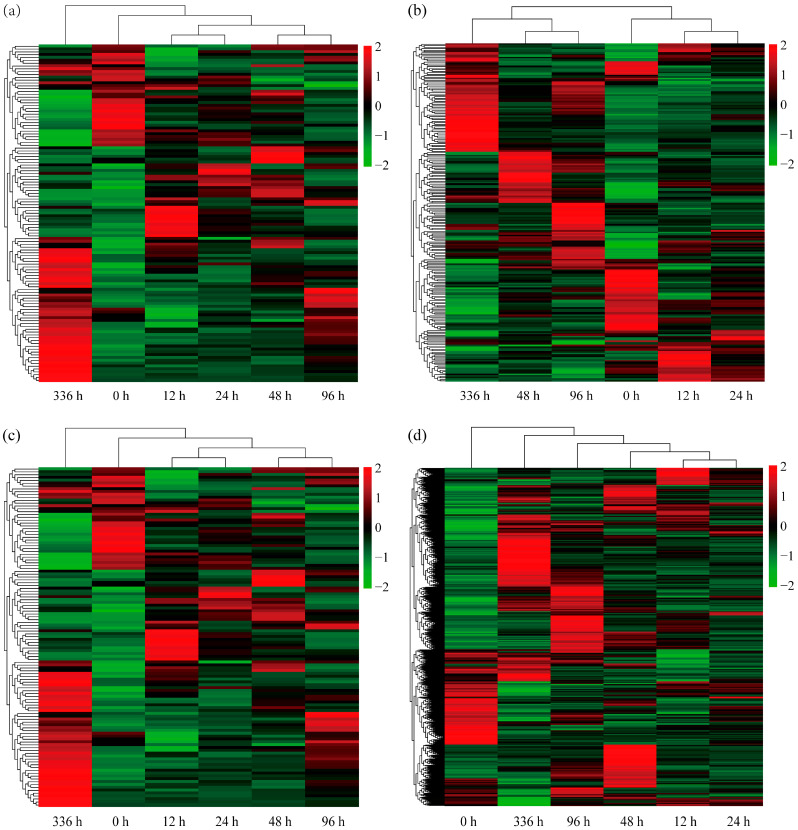
Heatmaps of differentially expressed genes (DEGs) in response to low-nitrogen (LN) conditions at different time points (0, 12, 24, 48, 96 and 336 h). (**a**) The clustering analysis of the expression patterns of DEGs related to nitrogen (N) metabolism under LN conditions at different time points. The x-axis represents time points, and the y-axis represents genes. Red indicates upregulated expression and green indicates downregulated expression. (**b**) The clustering analysis of the expression patterns of DEGs related to ROS scavenging under LN conditions at different time points. The x-axis represents time points, and the y-axis represents genes. Red indicates upregulated expression and green indicates downregulated expression. (**c**) The clustering analysis of the expression patterns of DEGs related to roots development under LN conditions at different time points. The x-axis represents time points, and the y-axis represents genes. Red indicates upregulated expression and green indicates downregulated expression. (**d**) The clustering analysis of the expression patterns of DEGs related to phytohormone under LN conditions at different time points. The x-axis represents time points, and the y-axis represents genes. Red indicates upregulated expression and green indicates downregulated expression.

After LN conditions, 113 DEGs related to N metabolism were identified (Table S3). To visualize the expression patterns of these N-metabolism-related DEGs across different treatment durations, a heatmap was generated (Figure 6a). The results showed rapid responses in some genes during early stress conditions (12 h and 24 h), with significant upregulation or downregulation. Other genes exhibited sustained increasing or decreasing trends at 48 h and 96 h. As stress extended to 336 h, more DEGs showed significant expression changes. Notably, we identified 21 DEGs involved in N uptake and transport (Table S3), including 14 genes encoding NRT1/PTR family (NPF) NRTs and 7 homologous genes encoding *AMT1/2* family ammonium transporters (AMTs). The *NPF6.4* (Potri.002G225500), *NPF3.1B* (Potri.010G126300), *NPF3.1D* (Potri.016G032000), *AMT1;2B* (Potri.002G255000), and *AMT2;1* (Potri.006G102800) genes were consistently significantly upregulated across all time points. Additionally, a sustained upregulation of the nitrate receptor *NLP7* (Potri.001G087900) gene and an SNF1-related protein kinase regulatory subunit beta-2 *SnRK1β2* (Potri.014G167400) gene were observed. Collectively, these genes may contribute to the adaptation of *P. ussuriensis* to LN conditions by participating in the regulation of N-absorption, transport, and assimilation processes.

### 3.5. Reactive Oxygen Species Scavenging-Related DEGs

ROS scavenging targets ROS that are produced during plant metabolism through th-e partial reduction of oxygen molecules. ROS can cause oxidative damage to cellular structures and components, inactivate proteases, and induce cell damage or death. Plants produce substantial ROS under abiotic stress conditions. To counteract inactivate proteases, plants employ an ROS scavenging system consisting of enzymes such as POD, catalase (CAT), glutathione S-transferase (GST), and superoxide dismutase (SOD).

This study identified 224 ROS-related DEGs under LN conditions, encompassing POD, CAT, GST (Table S4), and other genes. A heatmap (Figure 6b) visualized the expression patterns of these DEGs across treatment durations. The results demonstrated that during early stress (12–24 h), the expression levels of some genes were significantly increased; at mid-stress (48–96 h), new expression trends emerged; by late stress (336 h), early response genes’ expression declined, while new clusters were specifically induced or repressed. Further analyses revealed the consistent upregulation of five GST genes—such as *GST23A* (Potri.008G174900), *GST23B* (Potri.008G175100), *GSTU17* (Potri.010G032800), *GSTU45* (Potri.016G104500), and *GSTU8* (Potri.015G042000)—and three POD genes—such as *POD19* (Potri.004G052100), *POD4* (Potri.013G083600), and *PODN1* (Potri.016G125000)—under LN conditions at all time points (Table S4). We speculate that these genes may help maintain intracellular redox balance through the ROS scavenging system, assisting *P. ussuriensis* in adapting to LN conditions.

### 3.6. Root-Development-Related DEGs

Under LN conditions, 107 DEGs related to root development were identified (Table S5). To visualize dynamic changes in root-development-related DEGs across LN conditions, a heatmap was generated (Figure 6c). The results showed that during early LN conditions (12–24 h), some genes were significantly induced while others were suppressed; during middle-stage LN conditions (48–96 h), gene expression patterns diverged with increased variation, and early response genes either maintained stable expression or exhibited recovery trends; by the late stress phase (336 h), expression patterns stabilized, while differences from the early period measurements increased.

It is worth noting that under LN conditions, a gene related to root development regulatory *TCP9* (Potri.001G111800) and two genes related to lateral root development—*WRKY46* (Potri.002G168700) and *WRKY75* (Potri.015G099200)—were upregulated at all time points (Table S5). These findings suggest their potential role in the root development of *P. ussuriensis* under LN conditions.

### 3.7. Phytohormone-Related DEGs

This study identified 724 DEGs associated with eight phytohormone classes: auxin, cytokinin, abscisic acid, ethylene, gibberellin, jasmonate acid, salicylic acid, and strigolactone (Table S6). A heatmap visualizing the dynamic expression changes in these phytohormone-related DEGs across LN conditions is shown in Figure 6d. The results revealed that during early stress (12–24 h), some stress-responsive phytohormone genes were rapidly upregulated while others were significantly downregulated. During mid-stress (48–96 h), the expression of early responding genes stabilized as new clusters of upregulated or downregulated genes emerged. By late stress (336 h), synchronized upregulation and downregulation patterns suggested the establishment of LN adaptation homeostasis via phytohormonal signaling. Dynamic analysis across multiple time points further identified 48 phytohormone-related DEGs exhibiting sustained upregulation at all time points (Table S6). These primarily included 7 cytokinin-specific binding protein genes (all belonging to the Bet v 1 family), 4 gibberellin-related genes, 4 jasmonic acid-related genes, 5 salicylic acid-related genes, 8 abscisic acid-related genes, and 15 ethylene-related genes. Notably, both key ethylene biosynthesis *ACS2* (Potri.001G099400) and *ACO1* (Potri.011G020900) genes and two gibberellin regulatory protein *GASA1* (Potri.002G022500) and *GASA12* (Potri.017G124200) genes were significantly upregulated. The above results indicate that these genes may be involved in complex phytohormone signal transduction and regulatory processes, helping *P. ussuriensis* adapt to LN conditions.

### 3.8. Transcription Factor Families

**Figure 7:**
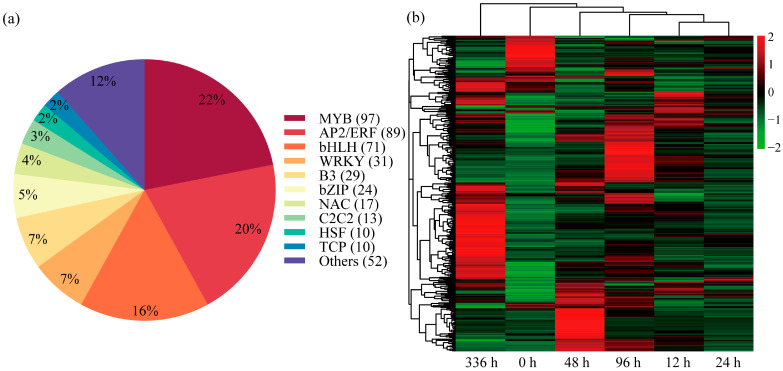
Analysis of transcription factors. (**a**) Composition and quantity of differentially expressed transcription factor families in the transcriptome of *Populus ussuriensis* under low-nitrogen (LN) conditions. (**b**) The heatmap of hierarchical clustering of transcription factor expression patterns across LN conditions time points. The horizontal axis represents time points, the vertical axis represents transcription factors, and colors indicate relative expression levels (red for upregulation; green for downregulation).

This study identified 443 differentially expressed TFs and classified them into 20 functional categories (Table S7). Among these, members of 10 major families (e.g., MYB, AP2/ERF, bHLH, WRKY, B3, bZIP, NAC, C2C2, HSF, TCP) accounted for 88% of all differentially expressed TFs (Figure 7a). Heatmap analyses of the LN condition time series (Figure 7b) revealed that the number of differentially expressed TFs at 96 h and 336 h was significantly higher than in the early stages, indicating substantial TF activation during the later stress phases. Further analysis revealed 33 genes consistently upregulated at all time points, belonging to the NAC, AP2/ERF, bHLH, WRKY, MYB, and TIFY families. In contrast, 11 genes were consistently downregulated at all time points, belonging to the bHLH, AP2/ERF, SBP, MYB, and bZIP families. To identify the most responsive candidates, we listed the top 10 most significantly upregulated and downregulated TFs based on their expression fold change in Table S8, which includes genes from the MYB, ERF, and bHLH families. To address the evolutionary conservation of nitrogen signaling, we analyzed the expression patterns of *P. ussuriensis* homologs of the key *A. thaliana* TGA transcription factors *AtTGA1* and *AtTGA4*. Under LN stress, all identified homologs—*TGA1A* (Potri.005G082000), *TGA1B* (Potri.007G085700), and *TGA4* (Potri.007G079900)—exhibited significant downregulation across all time points (12, 24, 48, 96, and 336 h), with Log_2_(FoldChange) values being consistently negative (Table S7). Notably, the suppression became more pronounced after 336 h of prolonged stress, suggesting a sustained repressive role of these TGA factors in the long-term adaptation to nitrogen limitation. Collectively, these results suggest that these TFs may play an important role in the complex regulatory network underlying *P. ussuriensis*’s response to low-nitrogen conditions.

### 3.9. Construction and Analysis of the N Metabolic Regulatory Network

**Figure 8:**
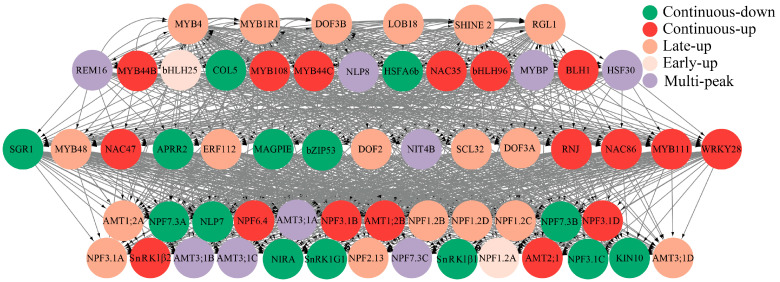
The construction of the nitrogen metabolism-related gene regulatory network in *Populus ussuriensis*. Circular nodes in the figure represent key genes involved in the nitrogen metabolism process of *P. ussuriensis* with gray connecting lines indicating regulatory relationships between genes, where the arrows point to the downstream genes. The hierarchical arrangement of nodes reveals the regulatory hierarchy, with each color representing a distinct layer, reflecting the complex transcriptional regulation and signaling network within the nitrogen metabolism pathway of *P. ussuriensis*.

This study employed a three-gene model-based GRN construction approach using the bottom-up GGM algorithm to reconstruct a putative GRN for N metabolism-associated DEGs from RNA-seq data (Figure 8). The resulting hierarchical GRN comprises four tiers with 60 nodes and 858 regulatory edges, organized into three functional classes: top-layer transcriptional regulators such as the continuously upregulated *MYB44B* (Potri.017G017600) and the early upregulated *bHLH25* (Potri.009G081400) genes; signaling hubs for signal integration and transduction, including the multi-peak expressed *NLP8* (Potri.007G133400), the continuously upregulated *NAC86* (Potri.001G144400), and the continuously upregulated *WRKY28* (Potri.005G203200) genes; and functional executors that mediate N physiology. The functional executions include the following: seven NRTs: the late-upregulated *NPF3.1A* (Potri.006G033900), the continuously downregulated *NPF3.1C* (Potri.006G034000), the continuously upregulated *NPF3.1B*, the continuously downregulated *NPF3.1D*, the continuously downregulated *NPF7.3A* (Potri.001G145200), the continuously downregulated *NPF7.3B* (Potri.003G088800), and the multi-peak expressed *NPF7.3C* (Potri.014G179400) genes; three AMTs: the late-upregulated *AMT1;2A* (Potri.019G023600), the continuously upregulated *AMT1;2B*, and the continuously upregulated *AMT2;1* genes; a nitrite reductase: the continuously downregulated *NIRA* (Potri.004G140800) gene; four carbon–nitrogen balancing kinases: the continuously downregulated *SnRK1G1* (Potri.012G097000), the continuously downregulated *SnRK1β1* (Potri.001G220800), the continuously upregulated *SnRK1β2*, and the continuously downregulated *KIN10* (Potri.013G090800) genes; and one assimilation activator: the continuously downregulated *NLP7*. Gray edges between nodes represent predicted activation or suppression relationships, collectively defining a complex, highly interconnected transcriptional regulatory architecture governing N response pathways.

### 3.10. Real-Time Quantitative Polymerase Chain Reaction (RT-qPCR) Validation

**Figure 9:**
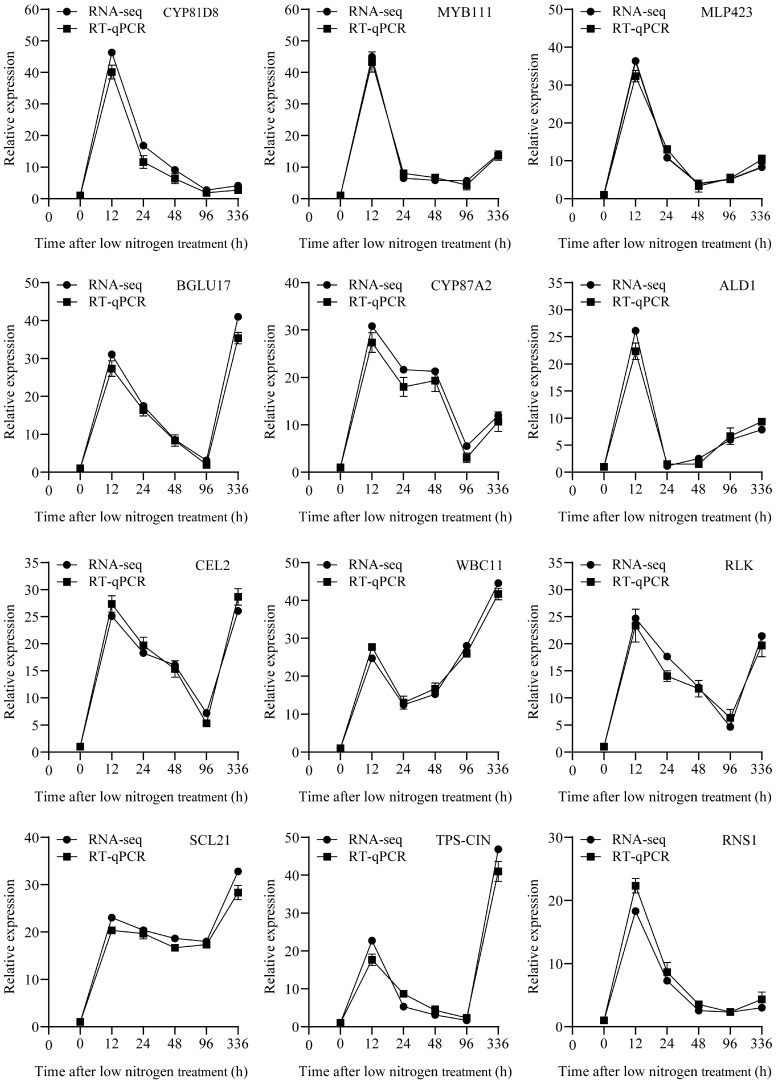
Real-time quantitative polymerase chain reaction (RT-qPCR) of different transcription factors under low-nitrogen conditions. The line graph presents the results of verifying the relative expression levels of 12 genes at different time points (0, 12, 24, 48, 96 and 336 h) using RNA sequencing and RT-qPCR assay. Each subpanel corresponds to the expression pattern of a specific gene. The black dots represent the RNA-seq data, and the black squares represent the RT-qPCR data. *Actin* served as the internal control. The experiment was performed with three biological replicates, values are mean ± SD (*n* = 3).

Twelve randomly selected genes were validated via RT-qPCR using *Actin* as the reference gene (Table S9). The expression patterns matched those from the RNA-seq results (Figure 9), confirming data reliability.

## 4. Discussion

Nitrogen is an essential macronutrient for plant growth and development. Under LN conditions, plants modulate their root morphology to enhance nutrient acquisition and meet N demands, with lateral root development being subject to significant N-dependent regulation [42]. In this study, we systematically characterized the dynamic responses of *P. ussuriensis* to prolonged LN conditions, integrating phenotypic measurements with transcriptome profiling across six time points (0, 12, 24, 48, 96, and 336 h). Our findings reveal a temporally coordinated reprogramming of gene expression networks that underlie root system remodeling, N acquisition and redistribution, redox homeostasis, phytohormone signaling, and transcriptional regulation, collectively enhancing plant acclimation to sustained N limitation.

### 4.1. Root Plasticity as a Primary Adaptive Strategy Under Nitrogen Deprivation

Nitrogen deficiency imposes a strong selective pressure on plant root systems, often triggering developmental reprogramming to enhance N acquisition from the soil. In this study, we observed a temporally distinct pattern of biomass allocation in *P. ussuriensis* under LN conditions. No significant changes were detected in either shoot or root biomass within the first 24 h under LN conditions. However, by 48 h, shoot fresh weight and plant height began to decline significantly, and this suppression intensified with prolonged stress duration. In contrast, root fresh weight increased significantly after 48 h of LN conditions and continued to rise through 336 h. These findings suggest a shift in carbon allocation favoring root growth over shoot development, consistent with classical nutrient foraging strategies observed in other species. Similar molecular mechanisms have been reported in *P. tremula* × *P. alba*, where a carbon partitioning shift is driven by a multi-tiered regulatory network. At early stages (6–24 h) of LN conditions, signal transduction pathways are activated. Subsequently, during the intermediate phase (48–96 h), the specific upregulation of cell cycle genes and DNA replication-associated proteins directly promotes root meristem proliferation. Ultimately, the sustained expression of root development genes optimizes root system architecture [43].

Transcriptome profiling further revealed the time-dependent modulation of root development-related genes in *P. ussuriensis*. During early stress (12–24 h), rapid changes in expression suggested that the root system was actively sensing N limitations. By mid-stress stages (24–96 h), these early response genes either stabilized or declined, while a distinct set of genes involved in root morphogenesis were differentially expressed. Upon prolonged stress (336 h), gene expression patterns stabilized, indicating a transition to a new homeostatic state. Notably, sustained upregulation was observed throughout LN for the transcription factor gene *TCP9* regulating root development, along with *WRKY46* and *WRKY75*, which stimulate lateral root development (Table S5). This expression pattern is consistent with their core function in stress responses. In *A. thaliana*, *TCP9* has been demonstrated to enhance tolerance to cyst nematode infection by mediating root system architecture remodeling [44]. Under ammonium stress conditions, *WRKY46* represses *NUDX9* and IAA-conjugating genes to maintain auxin homeostasis, thereby reducing ammonium efflux and promoting growth in the root elongation zone [45]. Furthermore, *WRKY75* enhances nutrient absorption efficiency by activating lateral root and root hair formation [46]. The findings indicate that under LN conditions, genes related to root development in *P. ussuriensis* exhibit spatiotemporally specific expression patterns.

The root preferential biomass allocation strategy observed in this study is analogous to the response of the gramineous crop rice under mild nitrogen deficiency. However, it contrasts with the strategy employed in some leguminous plants [47]. For instance, soybeans under nitrogen deficiency tend to rely more on symbiotic nitrogen fixation with rhizobia rather than significantly altering their root biomass allocation [48]. Research indicates that the key for maize in coping with low-nitrogen stress lies in its ability to recruit and maintain beneficial *Oxalobacteraceae* bacteria through the secretion of rhizosphere flavonoids instead of solely depending on increased root biomass [49]. This plant–microbe interaction triggers a positive feedback loop initiated by flavonoid recruitment, directly optimizing the plant’s nitrogen uptake efficiency. These differences highlight the evolutionary divergence among different plant groups in responding to low-nitrogen environmental stress.

### 4.2. Dynamic Regulation of N Transport and Metabolic Genes

Nitrogen acquisition and metabolism in plants involve a tightly coordinated network of processes, including uptake, transport, assimilation, and remobilization [47]. Higher plants primarily acquire N in the form of inorganic ions through specialized transporters localized on root cell membranes, including NRTs and AMTs [50]. Based on the transcriptome analysis conducted on *P. ussuriensis* under LN conditions, a significant enrichment of N metabolism genes was observed at most time points, except at 24 h (Figure 5), indicating a dynamic and time-dependent regulatory landscape. Differential expression analyses of N uptake and transport genes revealed diverse and temporally resolved expression patterns across multiple NRT family members (Figure 6a), with some genes exhibiting sustained induction or repression throughout the stress period while others responded transiently at distinct time points, reflecting a sophisticated transcriptional mechanism for tuning N acquisition in response to fluctuating nutrient availability. Notably, members of the NRT1/PTR (NPF) subfamily demonstrated functional divergence: *NPF6.4* was persistently upregulated, suggesting a role in high-affinity nitrate uptake analogous to the dual-affinity transporter *AtNRT1.1* in *A. thaliana* [51]. In contrast, *NPF7.3A* was consistently downregulated, potentially serving as an energy-conserving adaptation by limiting low-affinity nitrate transport under nutrient deprivation [52]. Additionally, *NPF3.1D* was significantly upregulated at 336 h (Table S3), implicating its involvement in late-stage adaptive processes such as root architectural remodeling or nitrate redistribution during prolonged stress. Concurrently, high-affinity AMTs (*AMT1;2B* and *AMT2;1*) were constitutively upregulated, aligning with functional validations where the overexpression of *BcAMT1.2* in rapeseed enhanced growth under low NH_4_^+^ conditions by mediating ammonium transport [53]. The function of *AMT2;1* in *P. trichocarpa* has also been verified [54].

Under LN conditions, *SnRK1β2* senses declining ATP/sucrose levels, activating the SnRK1 kinase complex, which phosphorylates the core N signaling factor *NLP7*, thus directly suppressing its transcriptional activity and inducing the sustained downregulation of its target gene nitrate reductase (*NIA*). This inhibition conserves energy by reducing nitrate assimilation while optimizing N acquisition efficiency [55]. These findings suggest that *P. ussuriensis* may adapt to N-deficiency conditions through the multi-level synergistic regulation of transporter activity, metabolic reprogramming, and energy allocation.

These dynamic transcriptional responses reveal a multi-level survival strategy in *P. ussuriensis*. High-affinity transporters such as *AMT1;2B* and *NPF6.4* are rapidly and persistently upregulated. This ensures a baseline capacity for nitrogen acquisition even under nitrogen scarcity. Simultaneously, certain NPF family members, including *NPF3.1D*, experience induction or suppression at specific stages. This expression shift indicates a transition in the plant’s strategy from short-term nitrogen uptake to long-term nitrogen management. This process may involve root system remodeling and internal nitrogen reallocation. This precise gene-level coordination is centrally regulated by the SnRK1-NLP7 signaling module. The module functions to balance nitrogen acquisition with the plant’s carbon and energy status. In *P. ussuriensis*, nitrate assimilation is reduced through the suppression of the nitrate reductase gene (*NIA)*, thereby conserving energy. Simultaneously, transporter activity is finely tuned. These coordinated actions form a synergistic adaptation mechanism that significantly enhances plant survival under prolonged nutrient stress. The results demonstrate that *P. ussuriensis* adapts to low-nitrogen conditions through multi-level synergistic regulation. This adaptation involves a network that dynamically and coordinately controls transport, metabolism, and energy allocation. These findings provide important insights into the molecular basis of nitrogen economy in perennial woody plants.

### 4.3. Fine-Tuned Redox Regulation Mediates Stress Adaptation

Nitrogen deficiency induces the excessive accumulation of ROS, which function dually as oxidative-damaging agents and secondary stress-signaling messengers [24]. In *P. ussuriensis*, LN conditions trigger a temporally dynamic response in ROS-scavenging genes in roots, exhibiting three characteristic phases—transient activation during the early stage (12–24 h), sustained regulation at the intermediate phase, and differential expression in the late phase (96–336 h)—and emphasizing the spatiotemporal complexity of the antioxidant network (Figure 6b).

Notably, five GST DEGs and three POD DEGs demonstrate persistent upregulation throughout the stress duration (Table S4). Cross-species comparisons confirm the core adaptive role of ROS-scavenging systems. For example, *O. sativa* enhances oxidative tolerance by inducing antioxidant enzymes, such as SOD, POD, and CAT [25]. Likewise, *Beta vulgaris* coordinately upregulates glutathione pathway components such as GST and GGT3 to reinforce ROS detoxification capacities [56]. These findings indicate that N deficiency activates the ROS-scavenging system in *P. ussuriensis*, protecting root cells from oxidative damage and thereby playing an important role in plant adaptation to LN conditions.

### 4.4. Hormonal Crosstalk Orchestrates Developmental and Metabolic Adjustments

Under LN conditions, phytohormones coordinate root development and NUE through a temporally regulated network, with signaling pathways significantly enriched at 12, 48, 96, and 336 h (Figure 5). *P. ussuriensis* roots exhibit a tri-phasic dynamic pattern of phytohormone-related gene expression, culminating in the establishment of a complex hormonal network supporting long-term adaptation (Figure 6d). Notably, 48 consistently upregulated DEGs across multiple hormone signaling pathways were identified (Table S6). The sustained upregulation of key genes within this network underscores their critical role. Particularly, the concerted induction of ethylene biosynthetic genes *ACS2* and *ACO1* suggests an enhancement of ethylene synthesis, while the activation of gibberellin-responsive genes *GASA1* and *GASA12* implies a role for gibberellin signaling. Collectively, these findings demonstrate that *P. ussuriensis* orchestrates long-term N stress adaptation through the temporally dynamic multi-stage regulation of phytohormone pathway gene expression. The coordinated action of key players, such as those in the ethylene and gibberellin pathways, provides foundational molecular insights into the hormonal mechanisms underlying prolonged N limitation tolerance.

### 4.5. Transcription Factor Networks Enable Stress Reprogramming

Under LN conditions, TFs function as core regulatory elements in plant stress responses, with their expression levels significantly increasing as stress duration extends to 96 and 336 h, indicating enhanced transcriptional activity during the later stages of stress. These differentially expressed TFs primarily belong to the MYB, AP2/ERF, bHLH, NAC, WRKY, B3, and bZIP families (Figure 7a). These TFs coordinate adaptive responses through synergistic regulation of downstream genes. For instance, heterologous expression in the rice of the foxtail millet (*Setaria italica*) R2R3-MYB TF gene *SiMYB30* demonstrated that the *SiMYB30* protein can directly bind to the proteins of the rice genes *OsGOGAT2*, *OsNRT1.1B*, and *OsNPF2.4* to activate their expression, thereby improving NUE in transgenic rice plants [57]. Concurrently, NAC and WRKY families regulate N transport and stress defense targets [58,59].

This study identified 44 core TFs, including 33 significantly upregulated and 11 significantly downregulated factors across eight families: NAC, AP2/ERF, bHLH, WRKY, MYB, TIFY, SBP, and bZIP. This persistent downregulation presents an intriguing contrast to the known role of their *A. thaliana* counterparts as positive regulators of the nitrate response. This discrepancy may suggest a species-specific functional divergence, where these TGA homologs in *P. ussuriensis* act as negative regulators to fine-tune the balance between growth and stress response under prolonged nitrogen deprivation. Alternatively, their repression could be part of a feedback mechanism to modulate the activity of other signaling pathways. This finding highlights the complexity and context-dependency of transcriptional regulation in nutrient stress adaptation. The coexistence of opposing regulatory polarities within key families (e.g., AP2/ERF, bHLH, MYB) confirms that TFs from different families play a central role in the complex regulatory network of *P. ussuriensis* in response to LN conditions, mediating the plant’s adaptation through precise synergistic regulatory mechanisms.

### 4.6. Structural Framework of N Metabolism GRN Reveals Transport-Centric Regulation

We constructed a putative GRN for DEGs associated with N metabolism. This network encompasses 21 gene families, including 14 classes of TFs (e.g., continuously upregulated *MYB44* and early upregulated *bHLH25*), 3 transporter families (NPF, AMT, and NRT), and 4 metabolic regulators (e.g., SnRK1, NLP). Analyses of expression dynamics revealed distinct transcriptional patterns among key regulators: signaling hubs such as *NLP8* showed multi-peak oscillations, while *NAC86* and *WRKY28* were continuously upregulated. Functional transporters also exhibited divergent responses. For instance, *AMT1;2A* was upregulated at later time points, whereas *NPF7.3B* and *NPF7.3C* were continuously downregulated. Notably, the NPF and AMT families collectively accounted for 42% of the network components, highlighting the pivotal role of N transport in this regulatory architecture. The predicted GRN provides a structural framework for investigating the molecular mechanisms underlying plant N response and assimilation. However, this proposed regulatory network remains hypothetical and requires further experimental validation through techniques such as using ChIP-seq, DAP-seq, EMSA, and Y1H assays to confirm the predicted interactions. The identified key gene functions from this transcriptome data provide a foundation for the subsequent cultivation of transgenic plants resistant to low-nitrogen condition.

## 5. Conclusions

This study aims to elucidate the response mechanism of *P. ussuriensis* root systems to LN conditions through phenotypic analysis and RNA-seq. Phenotypic observations revealed that after 48 h of LN conditions, WT in vitro plantlets exhibited a synergistic response characterized by increased root biomass and suppressed shoot growth. This phenotype intensified over time, indicating preferential resource allocation to roots. Transcriptome analysis identified 8289 DEGs, with significant expression differences in genes associated with N metabolism pathways, ROS scavenging pathways, root development pathways, and phytohormone signaling pathways. Focusing on the N metabolism pathway, a GRN for DEGs was constructed using a three-gene model framework. Network analysis revealed that multiple LN stress TFs, such as *NAC47* (Potri.019G031400), *NAC86*, and *MYB111*, mediate central regulatory roles in the N metabolism pathway: These TFs integrate upstream signals and coordinate plant adaptive responses to LN stress by activating downstream genes involved in uptake, transport, and assimilation. Collectively, temporal transcriptomics and N metabolism networks elucidate the regulatory complexity underlying *P. ussuriensis* root adaptation to sustained LN conditions across developmental stages.

## Data Availability

The raw sequence read from Illumina RNA sequencing has been uploaded to the GEO database (GEO accession: GSE306270).

## Supplementary Materials

The following supporting information can be downloaded at: https://www.mdpi.com/article/10.3390/biology14101448/s1, Table S1: Data filtering of the RNA sequencing in *Populus ussuriensis*; Table S2: Mapping rate between RNA sequencing in *Populus ussuriensis* and reference genome; Table S3: DEGs related to nitrogen in *Populus ussuriensis* under low-nitrogen condition; Table S4: DEGs related to ROS in *Populus ussuriensis* under low-nitrogen condition; Table S5: DEGs related to root development in *Populus ussuriensis* under low-nitrogen condition; Table S6: DEGs related to phytohormone in *Populus ussuriensis* under low-nitrogen condition; Table S7: Transcription factors response to low-nitrogen stress in *Populus ussuriensis*; Table S8: The top 10 most significantly upregulated and downregulated transcription factors in response to ​low nitrogen stress in *Populus ussuriensis*; Table S9: Primer design of Real-time quantitative PCR.

## Abbreviations

The following abbreviations are used in this manuscript:

GO	Gene Ontology
KEGG	Kyoto Encyclopedia of Genes and Genome
DEG	Differential gene expression
N	Nitrogen
ROS	Reactive oxygen species
POD	Peroxidase
CAT	Catalase
GST	Glutathione S-transferase
SOD	Superoxide dismutase
TF	Transcription factor
